# Impact of Notch3 Activation on Aortic Aneurysm Development in Marfan Syndrome

**DOI:** 10.1155/2022/7538649

**Published:** 2022-02-15

**Authors:** Kathryn Jespersen, Chenxin Li, Rishi Batra, Christopher A. Stephenson, Paul Harding, Kylie Sestak, Ryan T. Foley, Harrison Greene, Trevor Meisinger, Jason R. Cook, B. Timothy Baxter, Wanfen Xiong

**Affiliations:** ^1^Department of Surgery, University of Nebraska Medical Center, Omaha, NE 68198-7690, USA; ^2^Department of Ophthalmology, Shanghai Jiao Tong University, Shanghai, China

## Abstract

**Background:**

The leading cause of mortality in patients with Marfan syndrome (MFS) is thoracic aortic aneurysm and dissection. Notch signaling is essential for vessel morphogenesis and function. However, the role of Notch signaling in aortic pathology and aortic smooth muscle cell (SMC) differentiation in Marfan syndrome (MFS) is not completely understood.

**Methods:**

RNA-sequencing on ascending aortic tissue from a mouse model of MFS, *Fbn1^mgR/mgR^*, and wild-type controls was performed. Notch 3 expression and activation in aortic tissue were confirmed with real-time RT-PCR, immunohistochemistry, and Western blot. *Fbn1^mgR/mgR^* and wild-type mice were treated with a *γ*-secretase inhibitor, DAPT, to block Notch activation. Aortic aneurysms and rupture were evaluated with connective tissue staining, ultrasound, and life table analysis.

**Results:**

The murine RNA-sequencing data were validated with mouse and human MFS aortic tissue, demonstrating elevated Notch3 activation in MFS. Data further revealed that upregulation and activation of Notch3 were concomitant with increased expression of SMC contractile markers. Inhibiting Notch3 activation with DAPT attenuated aortic enlargement and improved survival of *Fbn1^mgR/mgR^* mice. DAPT treatment reduced elastin fiber fragmentation in the aorta and reversed the differentiation of SMCs.

**Conclusions:**

Our data demonstrated that matrix abnormalities in the aorta of MFS are associated with increased Notch3 activation. Enhanced Notch3 activation in MFS contributed to aortic aneurysm formation in MFS. This might be mediated by inducing a contractile phenotypic change of SMC. Our results suggest that inhibiting Notch3 activation may provide a strategy to prevent and treat aortic aneurysms in MFS.

## 1. Introduction

Marfan syndrome (MFS) is a dominantly inherited disorder of connective tissue with prominent abnormalities in the ocular, skeletal, and cardiovascular systems. The leading causes of mortality in patients with MFS are progressive aortic root dilation, aneurysm formation, and aortic dissection and rupture [1]. Although *β*-blockers have been the primary medical therapy for MFS patients with aortic aneurysms, current therapies for MFS have limited efficacy. Therefore, new approaches are required to identify novel therapeutic targets.

MFS is caused by mutations in the gene encoding fibrillin-1, a large extracellular matrix (ECM) glycoprotein. Fibrillin-1 is a major constituent of ECM microfibrils, which act as a scaffolding protein for elastin deposition and formation of elastic fibers [2–5]. It also binds or interacts with a large variety of ligands to regulate the cellular behavior and control cell survival, differentiation, and response to injury [6–14]. It has been demonstrated that fibrillin-1 binds to a disintegrin-like and metalloprotease with thrombospondin type-1 motif (ADAMTS) and ADAMTS-like (ADAMTSL) proteins to modulate microfibril assembly [6, 7], interacts with bone morphogenetic proteins (BMPs) and integrins to regulate cell function and differentiation [8–14], and interacts with latent TGF-*β* binding proteins (LTBPs) to regulate and control the local TGF-*β* bioavailability [15, 16]. The cardiovascular manifestations associated with fibrillin-1 gene (*FBN1*) mutations have been linked to elevated TGF-*β* signaling [15, 16]. Therefore, fibrillin-1 not only serves as a structure protein but also regulates local growth factor signaling. Through studying murine models of MFS, it has been shown that the haploinsufficiency caused by *FBN1* mutation plays an important role in the pathogenesis of MFS [17].

Notch signaling is essential for vessel morphogenesis and function. The notch genes encode large single-transmembrane receptors that mediate communication between neighboring cells that are crucial for cell fate decisions during organ development [18]. In mammals, four Notch receptors (Notch1–4) have been identified, all of which are composed of a large extracellular domain which mediates ligand interaction, a transmembrane domain, and an intracellular domain. Upon the binding of transmembrane ligands from Delta/Serrate/LAG-2 (DSL) family, DLL, and Jagged, Notch receptors undergo proteolytic cleavage by several proteinases, including *γ*-secretase [18]. This cleavage results in the release and translocation of the Notch intracellular domain (NICD) into the nucleus, where it interacts with the transcription factor CBF-1 to promote transcriptional activation and cell fate determination [19, 20]. In vascular smooth muscle cells (SMCs), the Notch2 and Notch3 receptors predominate [21–24]. Notch signaling is critical for the regulation of SMC contractile differentiation and extracellular matrix protein synthesis both *in vitro* and *in vivo* [21, 25, 26]. Studies by Crosas-Molist et al. showed that expression of SMC contractile protein markers was higher in the aortas of Marfan patients compared to healthy controls [27]. By using a murine model of MFS, *Fbn1^mgR/mgR^* mice, we have demonstrated that premature differentiation of SMCs contributes to aortic aneurysm pathology in MFS [28]. However, the role of Notch receptors in aneurysm formation and SMC differentiation in MFS remains unclear.

In this study, we observed increased Notch3 expression and activation in the aorta of *Fbn1^mgR/mgR^* mice. We investigated the impact of Notch3 in aortic aneurysm development in MFS. We found that inhibition of Notch signaling moderated VSMC contractile phenotype transition and attenuated aortic aneurysm development in *Fbn1^mgR/mgR^* mice.

## 2. Material and Methods

### 2.1. Mice

Heterozygous *FBN1* mutant mice (*Fbn1^mgR/+^*) in a mixed C57Bl/6J:129 SvEv background were mated to generate homozygous mutant mice (*Fbn1^mgR/mgR^*) and wild-type (WT) littermates [28]. Both male and female mice were included in the study. Genotyping of mice was performed at postnatal day 7 (PD7) by PCR [28, 29]. All experiments were carried out in accordance with the guidelines of the University of Nebraska Medical Center Animal Care Committee for the use and care of laboratory animals. All mice were maintained in the pathogen-free animal facility. Human TAA samples (*n* = 10) were provided from the NIH GenTac Biobank. Age- and gender- matched control aortic samples (*n* = 10) were obtained from Nebraska Organ Recovery System.

### 2.2. Next-Generation RNA-Sequencing and Real-Time RT-PCR

RNA from ascending thoracic aorta of WT and *Fbn1^mgR/mgR^* mice was extracted using TRIzol reagent (Thermo Fisher Scientific). The mRNA was converted to cDNA by reverse transcription, and sequencing adaptors were ligated to the ends of cDNA fragments. Following amplification by PCR, the RNA-Seq library was sequenced using an Illumina NextSeq500 sequence analyzer (Illumina, san Diego, CA). The sequence reads were processed with a series of software [30–32]. The values of fragments per kilobase of transcript per million mapped reads (FPKM) were used for statistical comparison. The significant differential expression genes were defined by *q* value ≤ 0.05 [33]. A list of normalized differentially expressed genes were visualized with heatmap.

For real-time RT-PCR, aortic RNA was reverse transcribed into cDNA using iScript Reverse Transcription Supermix (Bio-Rad Laboratories, Inc.). Real-time RT-PCR was performed using SsoAdvanced Universal SYBR® Green Supermix according to the manufacturer's instruction (Bio-Rad Laboratories, Inc.) on an ABI StepOne machine (Thermo Fisher Scientific). Fold differences were calculated using mRNA expression normalized to 18 s rRNA and analyzed using the *ΔΔ*Ct relative quantification method.

### 2.3. Immunohistochemistry and Verhoeff-Van Gieson (VVG) Connective Tissue Staining

After perfusion-fixation with 10% neutral-buffered formalin, mouse ascending aortic tissues were embedded in paraffin and cut into 4 *μ*m sections. For immunohistochemical staining, tissue sections were incubated with anti-Notch3 antibody (Santa Cruz Biotechnology, Dallas, TX) with a dilution of 1 : 250 for 32 min at 37°C. Discovery ChromoMap DAB kit (Roche, South San Francisco, CA) was used for antigen localization, and the slides were processed using Ventana Discovery Ultra instruments (Roche). For mouse tissue staining, microscopic fields (40X) of aorta were collected from each slide. Images were captured by Roche Ventana iScan HT (Roche). Definiens Tissue Studio software was used for the quantification of staining area and density. The mean density of Notch3 was determined for five regions of interest per aortic section. For human aortic sections, Notch3-positive cells were counted in each high power field (40x). The mean Notch3-positive cells were determined for five regions of each section. For VVG staining, mouse aortic sections were stained with Verhoeff's solution, ferric chloride, sodium thiosulfate, and Van Gieson's solution (Poly Scientific, Bay Shore, NY). Each staining cycle alternated between fixing and washing procedures. The slides were examined and photographed using light microscopy (20x; Nikon). The numbers of elastin breaks per field under 40x magnification were counted in each section. The mean values were determined for five regions of each section.

### 2.4. DAPT Treatment and Kaplan-Meier's Survival Curve

Beginning on PD10, WT (*n* = 10/group) and *Fbn1^mgR/mgR^* (*n* = 10/group) mice were treated with *γ*-secretase inhibitor, *N*-[*N*-(3,5-difluorophenacetyl)-l-alanyl]-*S*-phenylglycine t-butyl ester (DAPT) (Abcam, Cambridge, UK) until PD42. DAPT was suspended in DMSO. Mice were injected subcutaneously with 10 mg/kg of DAPT daily. A control group received DMSO only. Mice were randomly allocated in each group. WT littermates and *Fbn1^mgR/mgR^* mice without or with DAPT treatment were sacrificed at PD42. For histological studies, the ascending thoracic aortas were perfusion-fixed with 10% neutral buffered formalin and collected. For protein extraction, aortic samples were snap frozen in liquid nitrogen. To configure the Kaplan-Meier survival curves, mice (*n* = 15/group) were evaluated daily and survival recorded. Mice were followed up to 32 weeks, at which time all surviving mice were sacrificed.

### 2.5. High Frequency Ultrasound

Transthoracic ultrasound of WT and *Fbn1^mgR/mgR^* mice with or without DAPT treatment were performed with Vevo 3100 High Resolution In Vivo Micro-imaging system (VisualSonics, Toronto, Ontario, Canada) equipped with an integrated isoflurane-based anesthesia system. These studies were performed at 5, 8, and 12 weeks of age. Short-axis scans of the ascending aorta were performed using B-mode ultrasonography with the RMV 707 Scanhead. The aortic diameters were measured by M-mode in systole. Three independent measurements were obtained for each mouse. To define user variability, all echocardiographic studies were performed by 2 experienced individuals, and results were compared for agreement.

### 2.6. Isolation of Mouse SMC and Cell Culture

WT and *Fbn1^mgR/mgR^* mice (*n* = 6/group) were anesthetized and underwent laparotomy at PD28. Mouse thoracic aortas were isolated and minced. SMC isolation was described previously [28]. The cells were grown to confluence and passed after trypsinization with 0.25% trypsin. Aortic SMCs were maintained in vascular cell basal medium (ATCC, Manassas, VA) with 5% FBS. To examine the effect of DAPT, cells were incubated with serum-free medium and treated with 20 *μ*M DAPT for 48 h. DMSO treatment was used as vehicle control. Cells were harvested for protein extraction.

### 2.7. Western Blot Analysis

Aortic proteins were extracted as previously described [29]. Briefly, the protein from the aortic tissue and cells was extracted with RIPA lysis and extraction buffer (Thermo Fisher Scientific, Waltham, MA). The protein concentration of aortic proteins was standardized with a Bio-Rad protein assay (Bio-Rad Laboratories, Inc., Hercules, CA). Equal amounts (25-35 *μ*g) of aortic extracts from WT and *Fbn1^mgR/mgR^* mice without or with DAPT treatment were loaded into 4–20% Criterion TGX precast gels (Bio-Rad Laboratories, Inc.). Following electrophoresis, the gel was transferred onto a 0.45 *μ*m PVDF membrane (Bio-Rad Laboratories, Inc.). The membrane was incubated overnight at 4°C with antibodies directed against Notch3, *α*-actin, and *β*-tubulin (1 : 1,000) (Cell Signaling, Beverly, MA). Bound primary antibodies were detected with HRP-conjugated, species-specific, secondary antibodies (Cell Signaling) using the Clarity Western ECL system (Bio-Rad Laboratories, Inc.). The quantification was done using NIH ImageJ software and standardized by internal loading controls. The molecular sizes were determined using protein standards from Fermentas (Glen Burnie, MA).

### 2.8. Statistical Analyses

Data are presented as the mean ± the standard error (SE). Life table analysis was used for the Kaplan-Meier survival curve. For continuous variables, if the data were normally distributed, Student's *t*-test (comparison between two groups) or ANOVA with the appropriate post hoc test (comparison among groups of three or more) was used. Statistical significance was accepted at a *P* < 0.05.

## 3. Results

### 3.1. Notch3 Expression and Activation Were Increased in the Aorta of Both Humans and Mice with MFS

**Figure 1:**
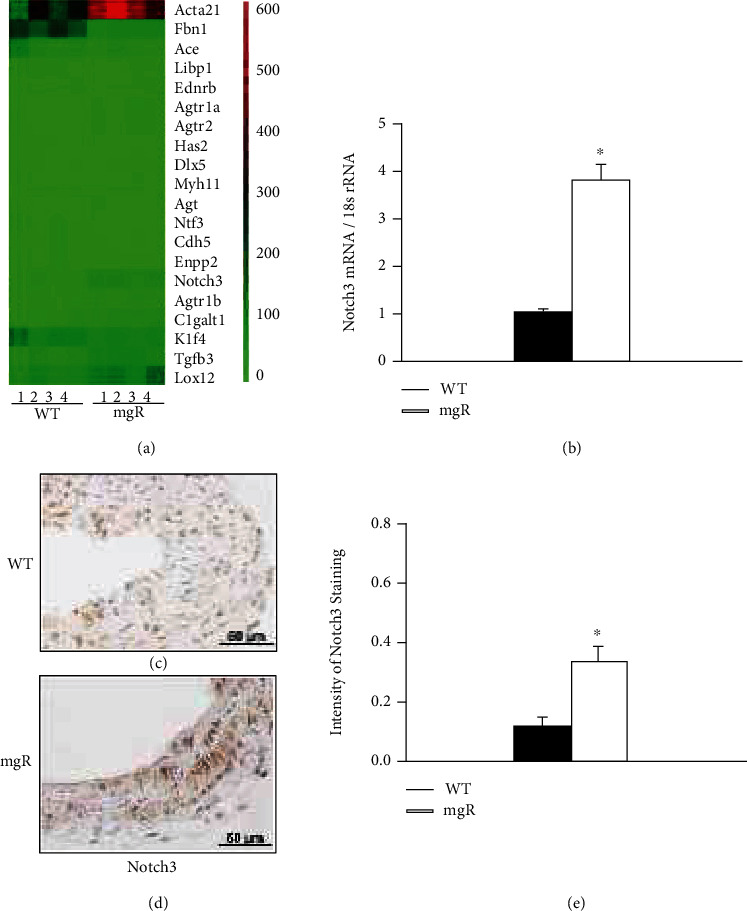
Genes were differentially expressed in the aorta of WT and *Fbn1^mgR/mgR^* (mgR) mice including Notch3, Fbn1, and *α*-actin. (a) Heatmap of the differentially expressed genes in the aorta of WT and mgR mice at PD28 (*n* = 4/group) was generated using data from RNA-Seq analysis. (b) Notch3 mRNA expression in the aorta of WT (*n* = 8) and mgR (*n* = 8) mice at PD28 was analyzed by real-time PCR. The bar graph (b) shows relative expression of Notch3 and 18 s rRNA. Immunohistochemical staining of Notch3 in the aortic sections from (c) WT and (d) mgR mice at PD28 (*n* = 4–6/group), respectively. Positive staining is shown in brown (DAB). Notch3-positive cells were quantitated by Definiens Tissue Studio software. Brown chromogen intensity is shown in (e) bar graph (*n* = 4–6/group). ^∗^*P* < 0.05 compared to WT controls; Student's *t*-test.

**Figure 2:**
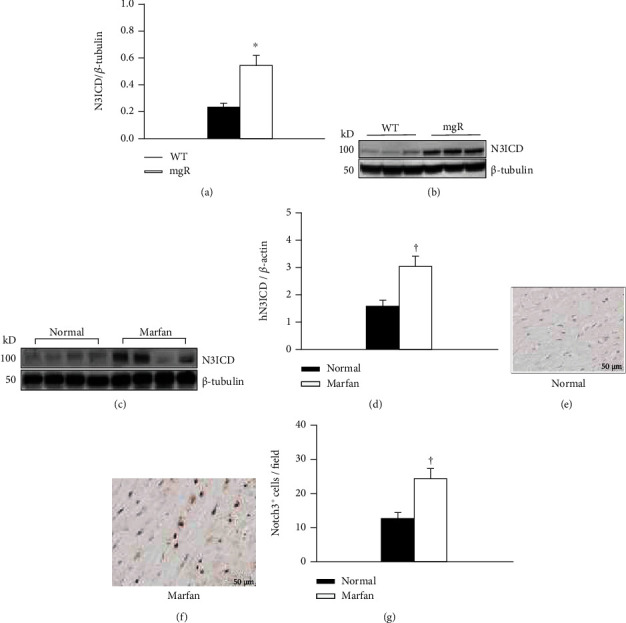
Notch3 activation was increased in the aorta of Marfan patients and *Fbn1^mgR/mgR^* (mgR) mice. Western blot analysis of active Notch3 (N3ICD) levels was performed on aortic protein from WT and mgR mice at PD28 (a) (*n* = 8/group) and Marfan patients and normal controls (*n* = 10/group) (c). The bar graphs show relative N3ICD levels in mouse (b) and human aortic tissue (d), respectively. ^∗^*P* < 0.05 compared to WT controls; ^†^*P* < 0.01 compared to normal controls; Student's *t*-test. Immunohistochemical staining of human Notch3 in the aortic sections from normal control (e) and Marfan patients (f) (*n* = 6/group). Positive staining is shown in brown (DAB). Notch3-positive cells were counted (cells/high power field, 40x). (g) The values reflect the mean ± SE. ^†^*P* < 0.01 compared to normal control; Student's *t*-test.

RNA-sequencing (RNA-Seq) analysis with aortic samples from WT and *Fbn1^mgR/mgR^* mice at PD28 (*n* = 4) was performed. The RNA-Seq data revealed an increased expression of Notch3 with upregulation of contractile proteins in *Fbn1^mgR/mgR^* mice (Figure 1(a)). This is consistent with our previous findings that aortic SMCs in MFS were prematurely differentiating into a contractile phenotype, i.e., upregulation of *α*-actin, SM22*α*, and calponin [28]. Increased Notch3 expression in the aortas of *Fbn1^mgR/mgR^* mice was confirmed with real-time PCR (Figure 1(b)). Immunohistochemical analysis of aortic tissue showed the protein levels of Notch3 were also increased in *Fbn1^mgR/mgR^* mice compared to WT controls at PD28 (Figures 1(c)–1(e)). Notch3 is a receptor that is composed of a large extracellular domain, a transmembrane domain, and an intracellular domain. Upon receptor-ligand binding at the cell surface, Notch3 undergoes proteolytic cleavage by proteinases, including *γ*-secretase [18]. This cleavage results in Notch3 activation and the release and translocation of the Notch3 intracellular domain (N3ICD) into the nucleus to promote transcriptional activation. We next tested activation of Notch3 in the aorta of WT and *Fbn1^mgR/mgR^* mice at PD28 by Western blot analysis. We found that active Notch3 (N3ICD) was significantly higher in *Fbn1^mgR/mgR^* mice compared to WT controls (Figures 2(a) and 2(b)). Furthermore, we sought to determine whether increased Notch3 activation is also seen in aortic samples from Marfan patients. The levels and activation of Notch3 in human aortic tissue were examined. Western blot analysis displayed that active Notch3 in the aorta was significantly higher in patients with MFS compared to matched controls (Figures 2(c) and 2(d)). Immunohistochemistry results showed that Notch3 levels were higher in Marfan patients than controls (Figures 2(e)–2(g)). These findings indicate that increased activation of Notch3 may play a role in the pathogenesis of human MFS and mouse model of MFS.

### 3.2. Inhibition of Notch3 Activation Preserved Elastic Fiber Integrity

**Figure 3:**
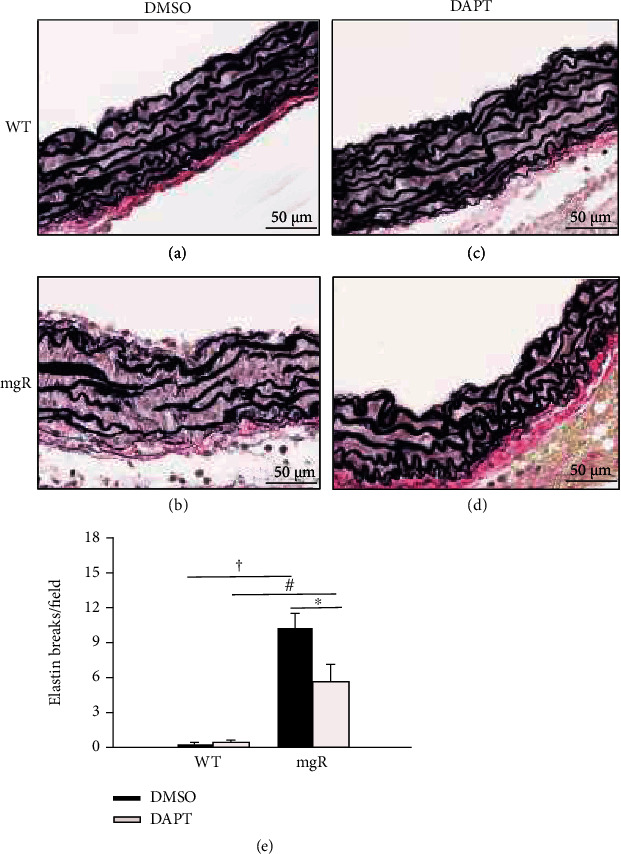
DAPT treatment, which inhibits Notch activation, prevented elastin fragmentation. (a–d) Verhoeff-van Gieson (VVG) staining of elastic fibers of the ascending aorta from WT and *Fbn1^mgR/mgR^* (mgR) mice at PD42 treated with DMSO (vehicle control) or DAPT; WT mice treated with DMSO (a) or DAPT (c); mgR mice treated with DMSO (b) or DAPT (d) at PD42. (e) Elastin breaks per field under 40x magnification in DMSO- or DAPT-treated WT and mgR mice (*n* = 5 aorta/groups). ^∗^*P* < 0.05, ^ǂ^*P* < 0.01, and ^†^*P* < 0.001, ANOVA with Tukey-Kramer post hoc test.

Previous studies of *Fbn1^mgR/mgR^* mouse aortas demonstrated aortic elastic fiber disorganization and fragmentation [28, 34]. To determine the role of Notch3 activation in aortic elastin degradation and aneurysm formation of *Fbn1^mgR/mgR^* mice, WT and *Fbn1^mgR/mgR^* mice were treated with a *γ*-secretase inhibitor, N-[N-(3,5-difluorophenacetyl)-L-alanyl]-S-phenylglucine t-butyl ester (DAPT). The treatment started at PD10 and stopped at PD42. The effects of DAPT treatment on aortic histology were evaluated by VVG staining. At PD42, while aortic lamellae in WT mice were intact and normal with treatment of DMSO or DAPT (Figures 3(a) and 3(c)), aortas of DMSO- (vehicle) treated *Fbn1^mgR/mgR^* mice showed elastic fiber disruption and medial hypertrophy (Figures 3(b)). However, DAPT-treated *Fbn1^mgR/mgR^* mice displayed a markedly lesser degree of aortic medial hypertrophy and elastin fragmentation (Figures 3(d) and 3(e)).

### 3.3. DAPT Treatment Delayed Aortic Expansion and Improved Survival of Fbn1^mgR/mgR^ Mice

**Figure 4:**
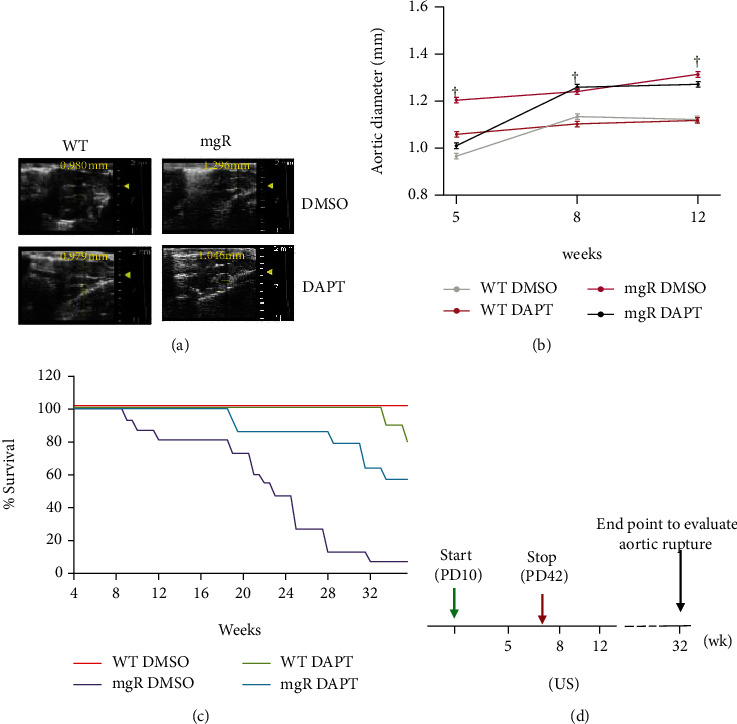
DAPT treatment delayed aortic expansion and improved survival of *Fbn1^mgR/mgR^* (mgR) mice. (a) Timeline of mouse experiments: WT and mgR mice were treated with DAPT (10 mg/kg) or DMSO by subcutaneous injection started at PD10 (start) and stopped at PD42 (stop). (b) Aortic diameters of mgR mice and WT littermates receiving DAPT or DMSO (*n* = 10/group) were measured by ultrasound (US) at 5, 8, and 12 weeks of age. The aortic diameters of DMSO-treated mgR mice were larger compared with WT controls at all time points (^†^*P* < 0.01 compared to WT controls). DAPT treatment inhibited aortic dilation of mgR mice at the 5-week time point (^∗^*P* < 0.01 compared to DMSO-treated mgR mice). (c) Representative ultrasound images of ascending aorta of mice at 5-week time point. (d) Life table analysis of WT (*n* = 10/group) and mgR (*n* = 15/group) mice treated with DAPT or DMSO from PD10 to PD42. Survival rate of mgR mice treated with DAPT was improved compared with DMSO-treated mgR mice (*P* < 0.01).

The ascending aortic diameters of WT or *Fbn1^mgR/mgR^* mice receiving DAPT or DMSO were measured with high-frequency ultrasound at 5, 8, and 12 weeks of age (Figure 4(a)). Aortic diameters of DMSO-treated WT mice were significantly smaller than those of DMSO-treated *Fbn1^mgR/mgR^* mice at all time points (Figure 4(b)). At the 5-week time point, aortic diameters in DAPT-treated *Fbn1^mgR/mgR^* mice were significantly smaller than those in DMSO-treated *Fbn1^mgR/mgR^* mice (Figures 4(b) and 4(c)). The aortic diameters in DAPT-treated mice did not differ from DMSO-treated mice at 8- or 12-week time points. However, by 8 weeks of age, 3 out of 10 *Fbn1^mgR/mgR^* DMSO-treated mice died from aortic rupture. Furthermore, survival studies were performed in WT and *Fbn1^mgR/mgR^* mice treated with DAPT or DMSO (Figure 4(d)). The mice were followed up until death or to 32 weeks. Mice surviving to 32 weeks were euthanized. WT mice survived a normal lifespan; 2 out of 15 WT mice treated with DAPT died after 30 weeks of age. The mean survival of DMSO-treated *Fbn1^mgR/mgR^* mice (*n* = 15) was 138.4 ± 13.6 days, while *Fbn1^mgR/mgR^* mice treated with DAPT (*n* = 15) survived 199.9 ± 10.5 days. This result demonstrated that DAPT treatment significantly prolonged the lifespan of *Fbn1^mgR/mgR^* mice. Taken together, these data suggest that inhibition of Notch3 activation could play a role in preventing the early developmental abnormalities that occur in the Marfan aorta.

### 3.4. DAPT Treatment Reversed Abnormal SMC Phenotypic Switch in Fbn1^mgR/mgR^ Mice

**Figure 5:**
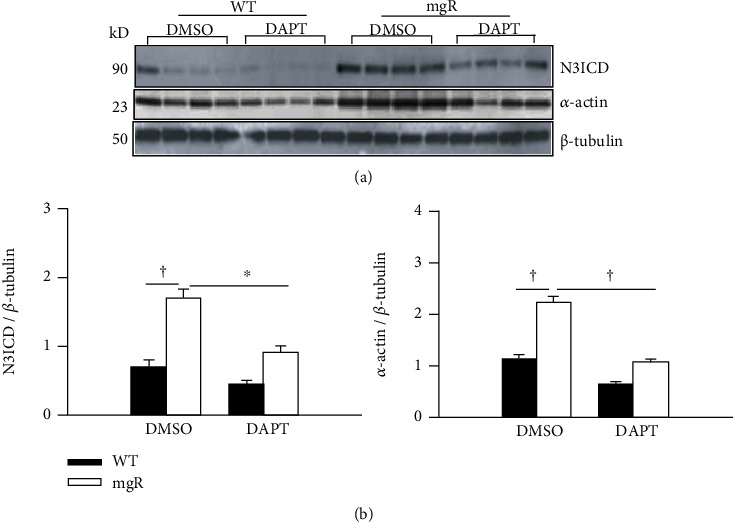
DAPT treatment blocked Notch3 activation and reversed SMC phenotype in the aorta of *Fbn1^mgR/mgR^* (mgR) mice. (a) Aortic protein from WT and mgR mice treated with DMSO and DAPT (*n* = 5-6/group) was extracted at PD 42. The levels of N3ICD and the smooth muscle cell differentiation marker, *α*-actin, were examined by Western blot analysis. (b) The bar graph shows relative active Notch3 (N3ICD) and *α*-actin levels in the aortic tissue. ^∗^*P* < 0.01 and ^†^*P* < 0.001; ANOVA with Tukey-Kramer post hoc test.

**Figure 6:**
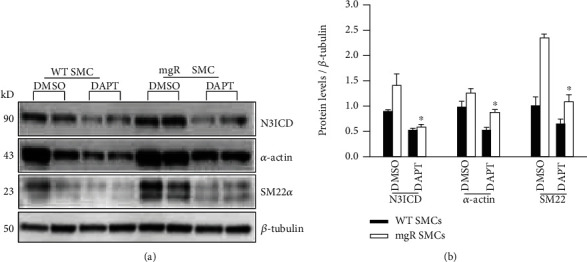
DAPT treatment inhibited Notch3 activation and reversed SMC phenotype of aortic SMCs from *Fbn1^mgR/mgR^* (mgR) mice. Aortic SMCs were isolated from WT and mgR mice and treated with DMSO or DAPT (20 mM) for 48 hrs. Protein from cells was extracted. (a) The levels of Notch3 activation (N3ICD), *α*-actin, and SM22*α* were examined by Western blot analysis. (b) The bar graph shows relative N3ICD, *α*-actin, and SM22*α* levels in the cells. ^∗^*P* < 0.05 compared to DMSO-treated mgR SMCs.

Our previous studies demonstrated that aortic SMCs of *Fbn1^mgR/mgR^* mice prematurely switched to a more contractile phenotype which contributed to aneurysm formation [28]. We sought to determine whether pharmacological inhibition of Notch3 activation in *Fbn1^mgR/mgR^* mice had effect on SMC differentiation. The levels of a SMC marker, *α*-actin, in the aorta were examined by Western blot (Figure 5(a)). While DAPT treatment inhibited aortic Notch3 activation in *Fbn1^mgR/mgR^* mice, it also reduced the expression of *α*-actin (Figures 5(a) and 5(b)). To confirm the effect of Notch3 activation on SMC differentiation, aortic SMCs were isolated from WT and *Fbn1^mgR/mgR^* mice. Consistent with the results from mouse aorta, N3CID levels were significantly higher in SMCs from *Fbn1^mgR/mgR^* mice than in SMCs from WT mice (Figures 6(a) and 6(b)). Furthermore, DAPT treatment reduced Notch3 activation and expression of contractile markers, *α*-actin and SM22*α*, in SMCs from *Fbn1^mgR/mgR^* mice. These results indicate that contribution of enhanced Notch3 activity to aneurysm formation in *Fbn1^mgR/mgR^* mice may be associated with aortic SMC differentiation. Taken together, these data suggest that inhibition of Notch3 activation could play a role in preventing the developmental abnormalities that occur in the Marfan aorta.

## 4. Discussion

We have shown that Notch3 expression and activation are increased in the aorta of MFS. During aortic development of MFS (*Fbn1^mgR/mgR^*) mice, increased Notch3 activation contributes to aortic aneurysm formation. Inhibition of Notch3 activation by *γ*-secretase inhibitor, DAPT, attenuates aortic elastic fiber fragmentation and aortic enlargement and improves mouse survival. The mechanism is due, in part, to mediation of aortic SMC phenotype modulation. These findings indicate that Notch3 is an important regulator of aortic SMC function and differentiation during aortic development and suggest that therapy modulating Notch3 signaling in the aorta may be beneficial in inhibiting aortic aneurysm formation in MFS.

The *Fbn1^mgR/mgR^* mice are one of commonly used murine models of MFS with a hypomorphic mutation of *FBN1*. Homozygous *Fbn1^mgR/mgR^* mice display clinical features and manifestations similar to classic patients of MFS. They die naturally at an average age of 2-3 months [34]. Previous studies showed that dysregulation of TGF-*β* activation contributed to pathogenesis of MFS [35, 36]. Pharmacological inhibition of TGF-*β* with TGF-*β*-neutralizing antibody attenuated aneurysm formation in MFS mice [16]. However, later results with the *Fbn1^mgR/mgR^* mouse model indicated that TGF-*β* could exert opposite effects on thoracic aortic aneurysm (TAA) pathology that broadly correlated with the early and late stages of TAA progression [37]. It was demonstrated that early treatment (PD16) with TGF-*β*-neutralizing antibodies exacerbated TAA formation, while later treatment (PD45) had a contrasting beneficial effect [37]. Our previous study also supported that initial consequence of *FBN1* mutation was not accompanied by a significant increase in TGF-*β* activation [28]. Differential contributions of TGF-*β* signaling to aortic physiology early and later after birth in MFS prompt us to identify new biomarkers and therapies independent of the TGF-*β* signaling pathway to better understand aortic pathogenesis in MFS.

It is demonstrated that aortic development in mice occurs primarily between embryonic day (ED)14 and postnatal day (PD)14 and is typically complete by PD28 [38, 39]. During this time, aortic SMCs are highly proliferative and express structural matrix proteins that are important for vascular strength and compliance. After that, aortic SMCs shift out of the matrix phase and express the spectrum of contractile proteins to prepare for their unique contractile function. In patients with MFS, aortic tissue and aortic SMCs had increased expression of contractile protein markers (*α*-actin, SM22*α*, and calponin-1) and collagen [27]. Our previous studies using a murine model of MFS, *Fbn1^mgR/mgR^* mice, supported these findings [28]. We demonstrated that aortic SMCs in MFS mice prematurely differentiated into a contractile phenotype, which contributed to the pathology in MFS [28]. We also showed that abnormal phenotypic switching of aortic SMC led to reduced elastin synthesis in Marfan mice [28]. However, the mechanisms modulating SMC phenotypic switching and matrix protein production in MFS are not completely understood. In this study, we have found that expression and activation of Notch3 are increased in the aortas of mice and humans with MFS, which is consistent with the findings from other investigators [40, 41]. Notch signaling is critical for cell growth regulation and cell fate determination in many cell types, including aortic SMCs [42, 43]. The regulation of contractile differentiation and extracellular matrix synthesis by Notch activity has been well studied, but results have shown that Notch signaling has both anti-differentiation and prodifferentiation functions in vascular SMCs, suggesting context dependence and/or spatial-temporal regulation during development [44–46]. The Notch signaling pathway plays an important role in the developing cardiovascular system. Notch1 is the primary expressed receptor in endothelial cells. Notch1 mutation is linked to bicuspid aortic valve and plays an important role in aortic root dilation [47, 48]. Notch2 and 3 are mainly expressed in vascular SMC and required for mural cell expansion and maturation. Notch4 is an endothelial cell-specific mammalian Notch gene [49]. In addition, Notch ligands, such as Jagged 1, also play important roles in normal arterial development [50–52].

Notch3 is predominantly expressed in vascular SMC in humans [25, 53–55]. It is a key regulator of vascular SMC phenotypes and is required for arterial identity and vascular SMC maturation [25, 53, 56]. Expression of the constitutively active Notch3 resulted in cell shape changes and an increase in *α*-actin [25]. Our studies showed similar results that levels of contractile protein, *α*-actin, were higher in the aorta of *Fbn1^mgR/mgR^* mice compared to control mice [28]. To determine the impact of increased Notch3 activity on the phenotypic state of VSMC in MFS, we administered DAPT, a *γ*-secretase inhibitor, to *Fbn1^mgR/mgR^* mice starting at PD10. DAPT inhibited Notch3 signaling by preventing the proteolysis of Notch3. DAPT treatment also reduced the expression of SMC contractile marker, *α*-actin, in the aorta of *Fbn1^mgR/mgR^* mice, indicating that DAPT treatment could reverse aortic SMC phenotype in *Fbn1^mgR/mgR^* mice. Furthermore, we investigated the effects of DAPT treatment on the aortic development and aneurysm formation/rupture of MFS. Blocking Notch3 activation with DAPT in the early aortic development of *Fbn1^mgR/mgR^* mice attenuated aneurysm enlargement and preserved elastic fiber integrity, therefore prolonging the lifespan of *Fbn1^mgR/mgR^* mice.

In summary, our data demonstrate that the early and potentially important SMC phenotypic switch and the associated matrix abnormalities in the Marfan aorta are associated with increased Notch3 activation. Increased Notch3 activation in MFS contributed to perturbation of early aortic development in MFS through inducing contractile phenotypic switch of SMC. The aortic pathology can be attenuated by treatment with a drug that blocks Notch3 activation. Our results suggest that inhibition of the effect of Notch3 signaling in the early aortic development of MFS may be a useful strategy to prevent and treat aortic aneurysms in MFS.

## Data Availability

The data used to support the findings of this study are available from the corresponding author upon request.

## Abbreviations

SMC: Aortic smooth muscle cell
MFS: Marfan syndrome
DAPT: *N*-[*N*-(3,5-Difluorophenacetyl)-l-alanyl]-*S*-phenylglycine t-butyl ester
ECM: Extracellular matrix.
